# Baoshentongluo Formula relieves podocyte injury in diabetic kidney disease through regulating mitophagy via PINK1/Parkin signaling pathway

**DOI:** 10.3389/fendo.2025.1606326

**Published:** 2025-05-23

**Authors:** Yanyu Pang, Lei Tian, Yufei Liu, Yifan Guo, Jingwen Zhao, Yutong Wang, Mengdi Wang, Wenjing Zhao

**Affiliations:** ^1^ Department of Nephrology, Beijing Hospital of Traditional Chinese Medicine, Capital Medical University, Beijing, China; ^2^ Dongfang Hospital, Beijing University of Chinese Medicine, Beijing, China; ^3^ Wangjing Hospital, Beijing University of Chinese Medicine, Beijing, China; ^4^ Beijing Hospital of Traditional Chinese Medicine, Beijing University of Chinese Medicine, Beijing, China

**Keywords:** diabetic kidney disease, traditional Chinese medicine, podocyte, apoptosis, mitochondria

## Abstract

**Introduction:**

Diabetic kidney disease (DKD) progression is strongly associated with podocyte mitochondrial dysfunction. The clinically effective Chinese herbal Baoshentongluo formula (BSTL) has demonstrated significant proteinuria reduction in DKD patients. HPLC-ESI-MS analysis identified characteristic bioactive components in BSTL including astragalosides, rehmanniosides, and tanshinones. However, the molecular mechanisms through which BSTL maintains podocyte homeostasis remain incompletely understood.

**Methods:**

Mouse podocyte clone-5 (MPC-5) cells and db/db mice were used. Db/db mice were randomized into db/db and db/db + BSTL (16.5 g/kg/d, intragastric administration for 12 weeks). A group of m/m mice served as the control. Renal function, urinary albumin-to-creatinine ratio (UACR), histopathological analysis, apoptotic, and mitophagy-related protein levels were evaluated. MPC-5 cells were exposed to high glucose (HG, 30 mM) and BSTL drug-containing serum (8%) for 24 h grouping as control, HG, HG + BSTL, and HG + siPINK1. Podocyte apoptosis, mitophagy levels, and expression of PTEN-induced putative kinase 1 (PINK1) and E3 ubiquitin ligase (Parkin) were assessed.

**Results:**

In db/db diabetic mice, oral administration of BSTL significantly lowered urinary albumin-to-creatinine ratio (P<0.05), improved glomerular filtration rate, and ameliorated renal histopathological changes, decreased LC3-II/LC3-I ratio, and downregulated expression of mitophagy-related proteins PINK1, Parkin, ATG5 and Beclin-1. Treatment with 8% BSTL-containing serum significantly attenuated HG-induced podocyte apoptosis (*P*<0.01) and suppressed excessive mitophagy, as evidenced by reduced TOM20/LC3 co-localization (P<0.01). Notably, BSTL treatment markedly reduced protein levels of both PINK1 and Parkin (P<0.01), key regulators of mitophagy initiation. Genetic silencing of PINK1 in podocytes phenocopied BSTL's protective effects, confirming the pathway specificity.

**Discussion:**

Our integrated in vitro and in vivo findings establish that BSTL protects against DKD progression by selectively inhibiting PINK1/Parkin-dependent mitophagy in podocytes to inhibit podocyte injury, which provides both mechanistic insights and therapeutic potential for clinical DKD management.

## 1 Introduction

Diabetic kidney disease (DKD) is a serious complication of diabetes, affecting approximately 40% of patients with type 2 diabetes and 30% of those with type 1 diabetes (1). As a leading contributor to chronic kidney disease (CKD), DKD substantially elevates patients’ risks of developing cardiovascular and cerebrovascular complications while significantly increasing mortality rates (2, 3). Current management strategies, including strict glycemic control, blood pressure regulation, and the use of renin-angiotensin system inhibitors (e.g., ACE inhibitors and ARBs), have demonstrated efficacy in slowing disease progression (4). However, these interventions often provide only partial protection, and many patients still experience declining renal function. Thus, there remains an urgent need for novel therapeutic approaches to more effectively halt or delay the progression of DKD.

Podocytes, situated in the outermost layer of the glomerular filtration membrane, are essential for maintaining the integrity and function of the glomerular filtration barrier. Under pathological conditions such as hyperglycemia, podocytes undergo structural alterations characterized by foot process effacement, widening, and constriction, which impair the glomerular filtration barrier’s integrity (5). When more than 20% of podocytes are lost due to detachment or apoptosis, this triggers irreversible pathological changes, including glomerulosclerosis and tubulointerstitial fibrosis, thereby exacerbating the progression of DKD (6). Consequently, podocyte injury has become a significant driver of proteinuria, glomerulosclerosis, and renal dysfunction in DKD (7–9). Furthermore, as terminally differentiated cells with limited regenerative capacity, podocytes play a critical role in maintaining glomerular filtration integrity. Consequently, protecting podocyte from injury represents a pivotal therapeutic strategy for slowing DKD progression (10).

In DKD, chronic hyperglycemia induces metabolic dysregulation, excessive reactive oxygen species (ROS) generation, and mitochondrial dysfunction, which cause podocyte structural and functional damage that accelerates disease progression (10). A substantial body of evidence highlights the central role of mitophagy in podocyte injury within DKD. As a specialized form of autophagy, mitophagy selectively targets mitochondria for degradation, ensuring mitochondrial quality control. Research has found that in the early stages of DKD, mitochondrial autophagy rates increase as a compensatory response to cellular stress. However, with disease progression, mitophagy becomes relatively insufficient, accumulating dysfunctional mitochondria within renal intrinsic cells, resulting in damage to these cells in the kidney. Notably, activating mitophagy can effectively mitigate mitochondrial dysfunction caused by external stressors when mitochondrial damage is within a compensatory threshold. Conversely, when mitochondrial damage exceeds this threshold, mitophagy becomes overactivated, leading to podocyte apoptosis. Therefore, modulating mitophagy represents a promising therapeutic strategy to mitigate podocyte injury and improve DKD outcomes.

The PINK1/Parkin pathway predominantly regulates ubiquitin-driven mitophagy, a critical process essential for maintaining mitochondrial function and initiating autophagic processes (11). This system comprises three key components: a mitochondrial damage sensor (PINK1), a signal booster (Parkin), and a signal executor (ubiquitin chain) (12, 13). Under physiological conditions, PINK1 is transported into the inner mitochondrial membrane via the translocase complex, where it is subsequently cleaved by the protease Parl1 (14, 15). However, when mitochondria are damaged, the loss of membrane potential prevents PINK1 from being translocated to the inner membrane, halting its degradation. This results in the accumulation of unprocessed PINK1 on the outer mitochondrial membrane, which recruits Parkin from the cytoplasm to dysfunctional mitochondria (16, 17). Notably, emerging evidence demonstrates that PINK1/Parkin-dependent mitophagy attenuates palmitic acid-induced podocyte apoptosis through suppression of mitochondrial ROS production. These findings underscore the therapeutic potential of targeting the PINK1/Parkin mitophagy pathway for protecting podocytes (18).

The unique multi-target effects of traditional Chinese medicine (TCM) on complex diseases have received increasing global interest and become a significant source for the discovery of new drugs in recent years (19–21). The Baoshentongluo formula (BSTL), consisting of seven herbs, including *Astragalus membranaceus*, *Rehmannia glutinosa*, *Salvia miltiorrhiza* Bunge, *Cuscuta chinensis* Lam, *Artemisia anomala* S. Moore, *Euonymus alatus* and *Hirudo nipponica*, has been firmly established as an effective treatment for DKD (22). Our prior HPLC-ESI-MS analysis identified key BSTL compounds—astragalosides, rehmanniosides and tanshinones (22). Existing research shows BSTL lowers urinary protein and slows DKD progression by protecting podocytes via AMPK-mediated mitochondrial biogenesis (22), but its precise protective mechanisms remain unclear. Using db/db diabetic mice and high glucose-treated podocytes, this study demonstrates BSTL’s protective mechanism against hyperglycemia-induced podocyte apoptosis and injury, potentially through the regulation of mitophagy, offering new therapeutic opportunities for diabetic kidney disease.

## 2 Materials and methods

### 2.1 Animals and treatment

The animals used for the experiment were 6-week-old male (n = 24) db/db mice and wild-type m/m mice obtained from Nanjing Institute of Biological Medicine (certificate number: SCXK2016-0010). All mice were housed in a facility maintained at a humidity of 60%, a constant temperature of 22-24°C, and a 12 h light/dark cycle. After 2 weeks of adaptive feeding, blood glucose levels were randomly measured in db/db mice, and mice were used in subsequent experiments when the blood glucose levels of mice exceeded 16.7 mmol/L on two occasions and the urinary albumin/creatinine ratio (UACR) was significantly elevated. The successfully generated db/db mice were randomly divided into a model group (db/db) and a BSTL group using the random number approach. The m/m mice were treated as the nondiabetic control group (con). Each group contained eight mice. The mice in the BSTL group received intragastric administration with 16.5 g/kg/d of crude BSTL based on the optimal concentration determined in previous studies by intragastric administration. BSTL was obtained from the Pharmacy of the Beijing Hospital of Traditional Chinese Medicine, Capital Medical University. The mice in the control and model groups were fed an equal volume of distilled water. Random blood glucose levels were tested every 2 weeks, and 8-h urine samples were collected from the mice every 4 weeks. After 12 weeks of treatment, all mice were anesthetized with sodium pentobarbital at 50 to 60 mg/kg. Meanwhile, we collected the serum of the mice, centrifuged at 4°C, 3000 rpm for 10 min, and stored at -80°C. After kidney extraction, the renal tissue was longitudinally sectioned into two halves: One half was fixed in 4% paraformaldehyde, followed by paraffin embedding for subsequent histopathological staining, immunohistochemistry, and immunofluorescence analyses. The other half was aseptically dissected to separate the cortex and medulla. The cortical portion was snap-frozen in liquid nitrogen and stored at −80°C for further protein extraction and western blotting. The mice were euthanized by decapitation. All animal experiments were performed in accordance with the protocol authorized by the Ethics Committee of the Beijing University of Chinese Medicine (BUCM-4-2020121804-4173).

### 2.2 Preparation of drug-containing serum

Eight-week-old male Sprague-Dawley (SD) rats (n = 40) weighing 200 ± 30 g were obtained from Beijing Huafukang Biotechnology Co., Ltd. After 1 week of acclimatization, rats were randomly divided into a blank group and a BSTL group (n = 20). Rats were fed a normal diet. The rats in the BSTL group were gavaged with BSTL solution at a dose of 36.6 g/kg/d, while the rats in the blank group were gavaged with the same volume of distilled water for 7 days. Before the final gavage, all rats fasted for 12 h. Then, they were anesthetized with sodium pentobarbital at a dose of 30 to 50 mg/kg 1 h after administration, and blood was collected from the abdominal aorta. The rats were euthanized by decapitation. The serum was deactivated at 56°C for half an hour, filtered, aliquoted, and stored at -80°C. All animal experiments were performed in accordance with the protocol authorized by the Ethics Committee of the Beijing Institute of Chinese Medicine (BJTCM-R-2025-03-01).

### 2.3 Cell culture and treatment

Mouse podocyte clone-5 (MPC-5) cells, identified by the NCBI taxonomy number 10090 and catalogued as CVCL_AS87 (iCell-m081), were used. For the *in vitro* experiments, MPC-5 cells were cultured at 33°C and 5% CO2 in Roswell Park Memorial Institute (RPMI) 1640 medium (11879020/11875093, Gibco, NY, USA) supplemented with 10% fetal bovine serum (FBS) (10099-141, Gibco), 100 µg/mL streptomycin, 100 U/mL penicillin G (V900929, Sigma, MO, USA), and 100 U/mL recombinant murine interferon (IFN)-γ (315-05-20, PeproTech, NJ, USA) for proliferation. After a culture period, podocytes were cultured at 37°C in RPMI 1640 medium without IFN-γ for 10–14 days to induce differentiation. The podocytes were used for subsequent experiments when the cell growth rate slowed, the cell volume significantly increased, and the foot processes expanded, indicating differentiation and maturation. Differentiated cells were cultured with normal glucose of 5.5 mmol/L (con), high glucose of 30 mmol/L (HG), and 30 mmol/L glucose with BSTL drug-containing serum (BSTL) for 24 h. The cells were collected for subsequent experiments.

### 2.4 PINK1 siRNA transfections

PINK1 siRNA (44599, Santa Cruz Biotechnology) was transfected into cells with the Lipofectamine^®^ RNAiMAX transfection kit (13778150, Invitrogen) according to the manufacturer’s protocol. Briefly, podocytes were cultured in 6-well plates for 24 h. The RNAiMAX transfection reagent and PINK1 siRNA were added to the reaction mixture. The podocytes were cultured with serum-free medium and reaction mixture for 24 h, and the medium was changed for 6–8 h. After transfection was completed, the podocytes were treated for 24 h with various media. The PINK1 gene level was measured by PCR to confirm the success of the transfection. Podocytes were collected 24 h after transfection for the following experiments.

### 2.5 Biochemical indicator measurements

The level of serum creatinine (Scr) and urinary creatinine was measured with a creatinine assay kit (C011-2-1, Nanjing Jiancheng Biotechnology, JiangSu, China) following the manufacturer’s instructions. A mouse albumin ELISA kit (ab108792, Abcam, OR, USA) was used to measure the level of urinary albumin. Urinary albumin and urine creatinine levels were used to calculate UACR.

### 2.6 Histological analysis of renal tissues

Renal tissues were immersed in 4% paraformaldehyde solution for 72 h, dehydrated, and embedded in paraffin. The kidney tissues were cut into 2-3 μm slices. Hematoxylin-eosin (HE), periodic acid-Schiff (PAS), and Masson staining were performed by the Department of Pathology, Beijing Hospital of Traditional Chinese Medicine, Capital Medical University. The stained sections were observed with a Leica microscope (Aperio CS2, Germany). Representative kidney tissue structures were selected and photographed.

### 2.7 Western blot

Renal cortex tissues and MPC-5 cells were lysed with radioimmunoprecipitation (RIPA) lysis buffer (1:50, C1053, Applygen, Beijing, China) supplemented with protease inhibitors (1:100, P1260, Applygen). A bicinchoninic acid (BCA) protein quantification kit (P1511, Applygen) was used to quantify the protein in the samples. The protein extract was boiled at 95°C for 15 min. Samples from different groups (15 to 20 μg per well) were electrophoresed on polyacrylamide gels, which were electrotransferred to polyvinylidene difluoride (PVDF) membranes. After protein transfer, 5% skim milk was used to block the membranes for 1 h. The membranes were incubated at 4°C overnight with anti-Bcl-2 (1:2000, ab182858, Abcam), anti-Bax (1:5000, 50599-2-AP, Proteintech, Wuhan, China), anti-ATG5 (1:2000, 66744-1-Ig, Proteintech), anti-Beclin-1 (1:2000, ab207612, Abcam), anti-LC3B (1:2000, ab192890, Abcam), anti-PINK1 (1:500, ab23707, Abcam), anti-Parkin (1:1000, ab77924, Abcam) and protein loading control of anti-glyceraldehyde phosphate dehydrogenase (GAPDH, 1:5000, 10494-1-AP, Proteintech) primary antibodies and then incubated with secondary antibodies at room temperature for 1 h. Final detection was performed using enhanced chemiluminescence (ECL) hypersensitive substrate. Protein bands were primarily captured with a digital imaging system, while X-ray film was employed in **Figure 5B**.

### 2.8 Immunohistochemistry

Embedded kidney tissue was sectioned (2-3 μm). The sectioned tissues were baked at 60°C for 1 h, dewaxed with xylene three times for 45 min, hydrated with a gradient ethanol solution, and washed with deionized water. Subsequently, renal sections were immersed in the antigen retrieval solution, placed in a 95°C water bath for 20 min for antigen retrieval, and allowed to cool naturally to room temperature. Incubation with 3% H2O2 was used to quench the endogenous peroxidase activity. The renal sections were blocked with goat serum (ZLI-9056, ZSBIO company, Beijing, China) at 37°C for 30 min and incubated with anti-PINK1 (1:500, ab23707, Abcam) and anti-Parkin (1:400, 14060-1-AP, Proteintech) antibodies overnight at 4°C. The next day, the sections were washed with phosphate-buffered saline (PBS) three times, incubated with horseradish peroxidase-conjugated anti-rabbit secondary antibody (PV-9001, ZSBIO business), and developed with diaminobenzidine (DAB, ZLI-9018, ZSBIO company). Finally, the nuclei were counterstained with hematoxylin. A Leica microscope (Aperio CS2, Germany) was used to observe the kidney sections.

### 2.9 Immunofluorescence

The embedded renal sections were dewaxed, hydrated, and subjected to antigen retrieval as described for IHC. The renal sections were permeabilized with a 0.3% phosphate-buffered solution containing Tween-20 (PBST) and blocked with 3% donkey serum. Next, the renal sections were incubated with a mixture of rabbit anti-LC3B (1:200, ab192890, Abcam) antibody and mouse anti-TOM20 (1:50, sc17764, Santa Cruz Biotechnology) antibody overnight at 4°C. Renal sections were stained with Alexa Fluor 488-conjugated donkey anti-mouse IgG (1:2000, A21206, Invitrogen, PA, USA) or a mixture of Alexa Fluor 488-conjugated donkey anti-mouse IgG (1:2000, A21206, Invitrogen) and Alexa Fluor 594-conjugated donkey anti-rabbit IgG (1:2000, A32754, Invitrogen) as secondary antibodies at 37°C for 1 h after washing with PBS. The nuclei were counterstained with 4’,6-diamidino-2-phenylindole (DAPI, ZL1-9557, ZSBIO company). Sections were observed by fluorescence microscopy (A1 HAL 100, ZEISS Scope, Germany) or laser scanning confocal microscopy (LSM 800, ZEISS, Germany).

*In vitro*, MPC-5 cells were cultured in 12-well plates with different media (con, HG, BSTL, and siPINK1) for 24 h after reaching 80% confluence. The podocytes were immersed in 4% paraformaldehyde. After permeabilization with 0.3% Triton X-100 and blocking with 3% donkey serum, podocytes were incubated with a mixture of rabbit anti-LC3B (1:200, ab192890, Abcam) antibody and mouse anti-TOM20 (1:50, sc17764, Santa Cruz Biotechnology) antibody overnight at 4°C. After washing with PBS, the podocytes were treated with a mixture of Alexa Fluor 488-conjugated donkey anti-mouse IgG (1:2000) and Alexa Fluor 594-conjugated donkey anti-rabbit IgG (1:2000) as secondary antibody at 37°C for 1 h, and the nuclei were counterstained with DAPI. A laser scanning confocal microscope (LSM 800, ZEISS, Germany) and a fluorescence microscope (A1 HAL 100, ZEISS Scope, Germany) were used to observe the cells.

### 2.10 Terminal deoxynucleotidyl transferase-mediated dUTP-biotin nick end labelling analysis

*In vivo*, the Dead-End™ Colorimetric TUNEL System (G7130/G7160, Promega, WI, USA) was used to detect apoptotic glomerular cells. The embedded renal sections were dewaxed and hydrated as described for IHC. The renal sections were immersed in 4% paraformaldehyde, incubated with proteinase K, immersed in 4% paraformaldehyde again after being washed with PBS, and equilibrated for 10 min with an equilibration buffer. Renal sections were treated with an R-terminal deoxynucleotidyl transferase (rTdT) reaction mixture and immersed in 2X saline sodium citrate (SSC). The renal sections were incubated with 0.3% hydrogen peroxide after washing with PBS, treated with streptavidin horseradish peroxidase (HRP) solution, and developed with DAB. Finally, the renal sections were sealed with 100% glycerin and observed using a Leica microscope (Aperio CS2, Germany).

*In vitro* podocyte apoptosis was assessed with an *in situ* cell death detection kit (11684817910, Roche, BASEL, SWZ). MPC-5 cells were cultured in 6-well plates with different media according to the groups for 24 h. The cells were treated with the TUNEL reaction mixture at 37°C for 1 h, washed with PBS, and counterstained with DAPI. A fluorescence microscope (CKX41, OLYMPUS) was used to detect apoptotic podocytes.

### 2.11 RNA extraction and real-time PCR analysis

Total RNA was extracted from cultured cells using TRIzol (15596-018, Invitrogen, Carlsbad, CA, USA) and reverse transcribed into cDNA. Real-time PCR was performed using SYBR Green reagent on a Bio-Rad CFX PCR System (Bio-Rad, Carlsbad, CA, USA).

### 2.12 Flow cytometry

Podocytes were cultured in 6-well plates and treated with different media for 24 h. The level of podocyte apoptosis was measured using an Annexin V-fluorescein isothiocyanate (FITC) apoptosis detection kit (556547, Becton Dickinson and Company, NY, USA). Briefly, podocyte density was adjusted to 1 × 106 cells/mL, and 5 μL Annexin V-FITC and 5 μL propidium iodide (PI) were added to 100 μL cell suspensions. After incubation at room temperature for 30 min in the dark, podocytes were centrifuged and resuspended in a binding buffer. A flow cytometer (Calibur II, Becton, Dickinson, and Company, USA) was used to analyze the podocytes from the different groups. FlowJo software was used to analyze the Annexin V-FITC/PI levels.

### 2.13 Statistical analysis

All data were analyzed using IBM SPSS 26.0 software. The normality of distribution was assessed using the Shapiro-Wilk test, while homogeneity of variance was evaluated through both Bartlett’s and Levene’s tests. For data meeting both normality and homogeneity assumptions, one-way ANOVA was employed, followed by Dunn-Bonferroni *post hoc* tests for intergroup comparisons. When variances were unequal, Welch’s ANOVA was applied. Non-normally distributed data were analyzed using the nonparametric Kruskal-Wallis test. A p-value < 0.05 was considered statistically significant. Data visualization was performed using GraphPad Prism version 7.0.

## 3 Results

### 3.1 BSTL improved renal function and attenuated renal histological damage in db/db mice

**Figure 1:**
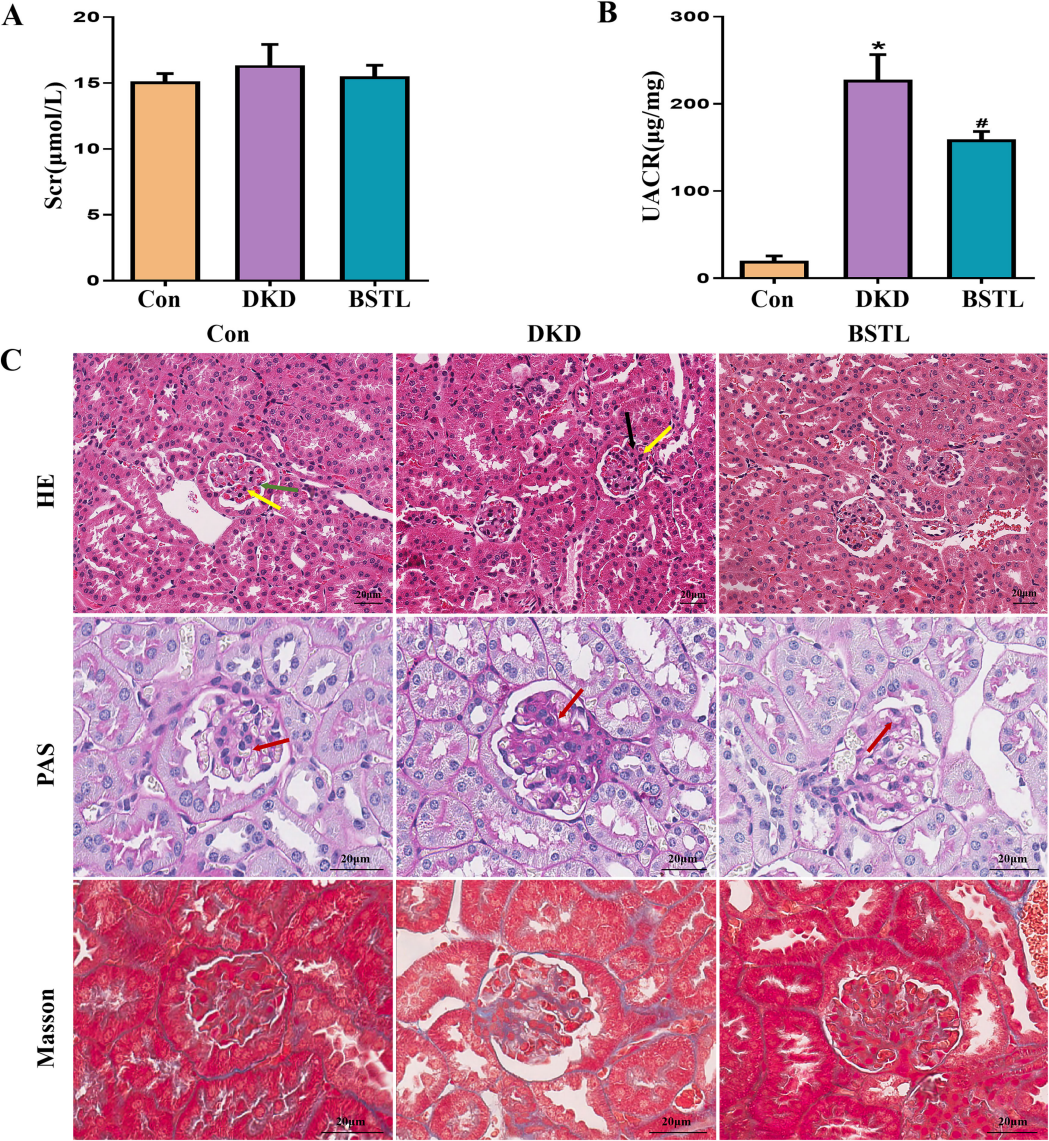
BSTL treatment improved renal function and attenuated renal histological damage in db/db mice. **(A)** Quantitative assessment of Scr in mice. **(B)** Quantitative assessment of UACR in mice. **(C)** Representative micrographs of HE-stained kidney sections (×200), PAS-stained kidney sections (×400), and Masson’s trichrome-stained kidney sections (×400) from different groups. Red arrows: glomerular basement membrane; green arrows: endothelial cells; yellow arrows: mesangial cells; black arrows: mesangial matrix. *P<0.05 *vs*. con; #P<0.05 *vs*. DKD. con, control mice; DKD, db/db mice; BSTL, db/db mice treated with BSTL.

By six weeks of age, db/db mice, an established model for type II diabetes mellitus, exhibited marked obesity and elevated fasting blood glucose levels, peaking between 8 and 12 weeks. To verify the efficacy of BSTL in db/db mice, at the end of 12 weeks of treatment, we collected blood and urine samples from mice and measured 8-hour urinary albumin, urinary creatinine, and serum creatinine levels. As shown in **Figures 1A, B**, db/db mice exhibited significantly higher UACR compared to control mice, along with elevated serum creatinine levels that did not reach statistical significance. In contrast, BSTL treatment markedly reduced UACR and partially improved Scr levels in db/db mice, indicating a protective effect on renal function.

To evaluate the impact of BSTL on renal pathology in db/db mice, we performed HE, PAS, and Masson staining on renal tissue sections from each group, examining pathological changes by optical microscopy. In the control group, the glomeruli exhibited a well-defined structure with a thin basement membrane and no increase in endothelial cells, mesangial cells, or mesangial matrix, characteristics typical of normal renal architecture. In contrast, db/db mice showed marked glomerular basement membrane thickening and increased mesangial matrix density. Notably, BSTL treatment significantly mitigated these pathological alterations (**Figure 1C**).

### 3.2 BSTL inhibited podocyte injury and apoptosis in db/db mice and HG-cultured podocytes

**Figure 2:**
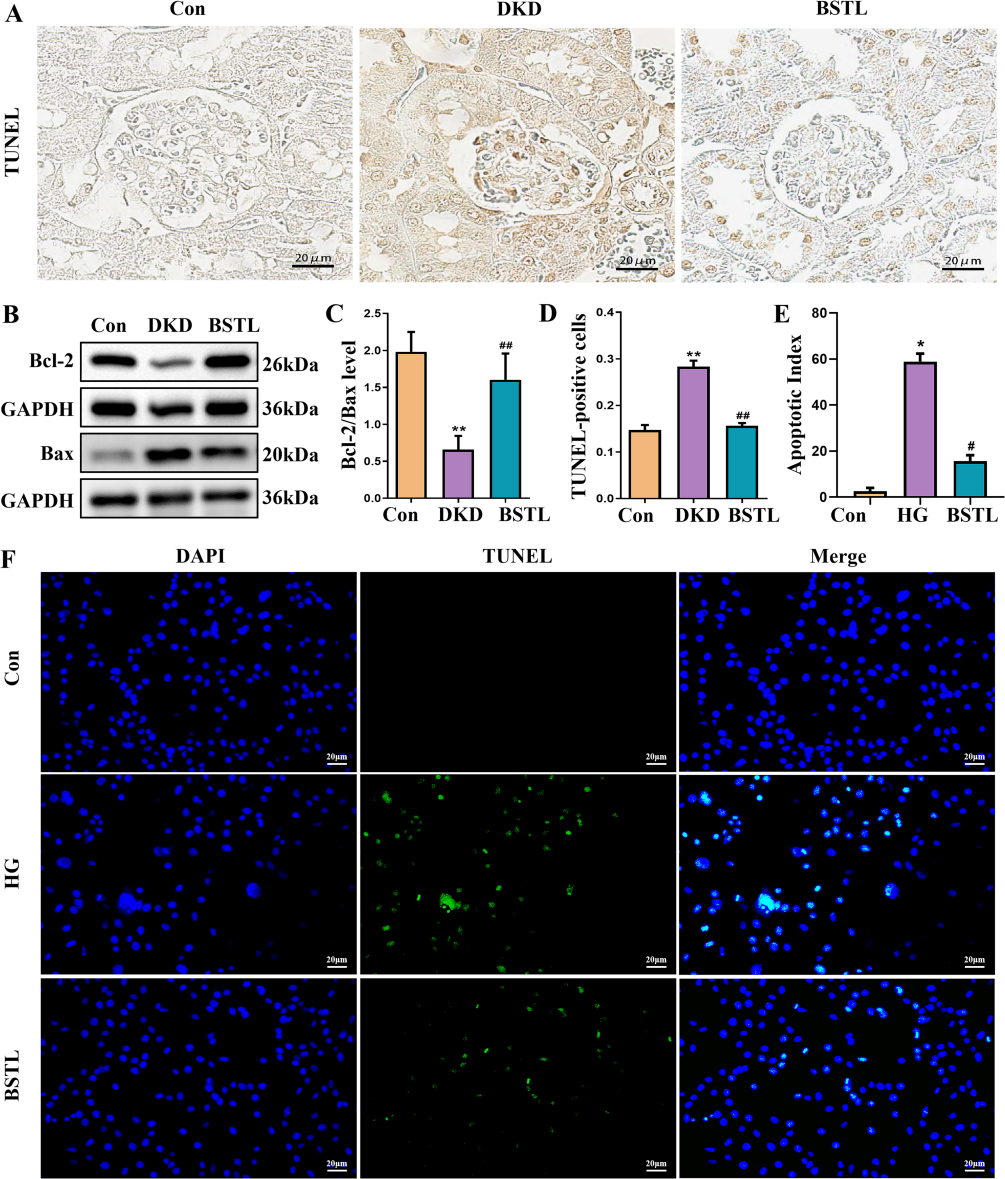
BSTL treatment inhibited podocyte injury and apoptosis in db/db mice and HG-treated podocytes. **(A)** TUNEL staining in renal sections from different groups (×400). **(B)** Representative Western blot of Bcl-2 and Bax in the kidneys of mice. **(C)** Quantitative assessment of Bcl-2 and Bax in the kidneys of mice. **(D)** Quantitative assessment of TUNEL staining in renal sections of different groups. *P<0.05 *vs*. con; **P<0.01 *vs*. Con; #P<0.05 *vs*. DKD; ##P<0.01 *vs*. DKD. con, control mice; DKD, db/db mice; BSTL, db/db mice treated with BSTL. **(E)** Quantitative assessment of TUNEL staining in podocytes from different groups. **(F)** TUNEL staining in podocytes of different groups (×200). *P<0.05 *vs*. con; #P<0.05 *vs*. HG. con, normal glucose; HG, high glucose; BSTL, high glucose combined with BSTL drug-containing serum.

Expression levels of Bcl-2 and Bax serve as indicators of apoptosis, and the reduction of podocyte apoptosis is a key therapeutic approach for DKD. To evaluate the efficacy of BSTL, we analyzed Bcl-2 and Bax expression. Compared to the control group, db/db mice exhibited reduced Bcl-2, elevated Bax, and a lower Bcl-2/Bax ratio, reflecting increased apoptosis. BSTL treatment effectively reversed these changes (**Figures 2B, C**). TUNEL staining was also performed to assess glomerular cell apoptosis in renal tissues, producing consistent results (**Figures 2A, D**). To further explore anti-apoptotic effect of BSTL on podocytes, we conducted *in vitro* experiments with MPC-5 cells exposed to high glucose. TUNEL staining confirmed that high-glucose conditions significantly increased apoptosis in MPC-5 cells, while BSTL treatment markedly reduced the number of apoptotic cells (**Figures 2E, F**). These findings suggest that BSTL effectively mitigates podocyte damage, supports filtration barrier integrity, and reduces proteinuria.

### 3.3 BSTL reduced the expression of mitophagy-associated proteins in renal tissues of db/db mice and HG-treated podocytes

**Figure 3:**
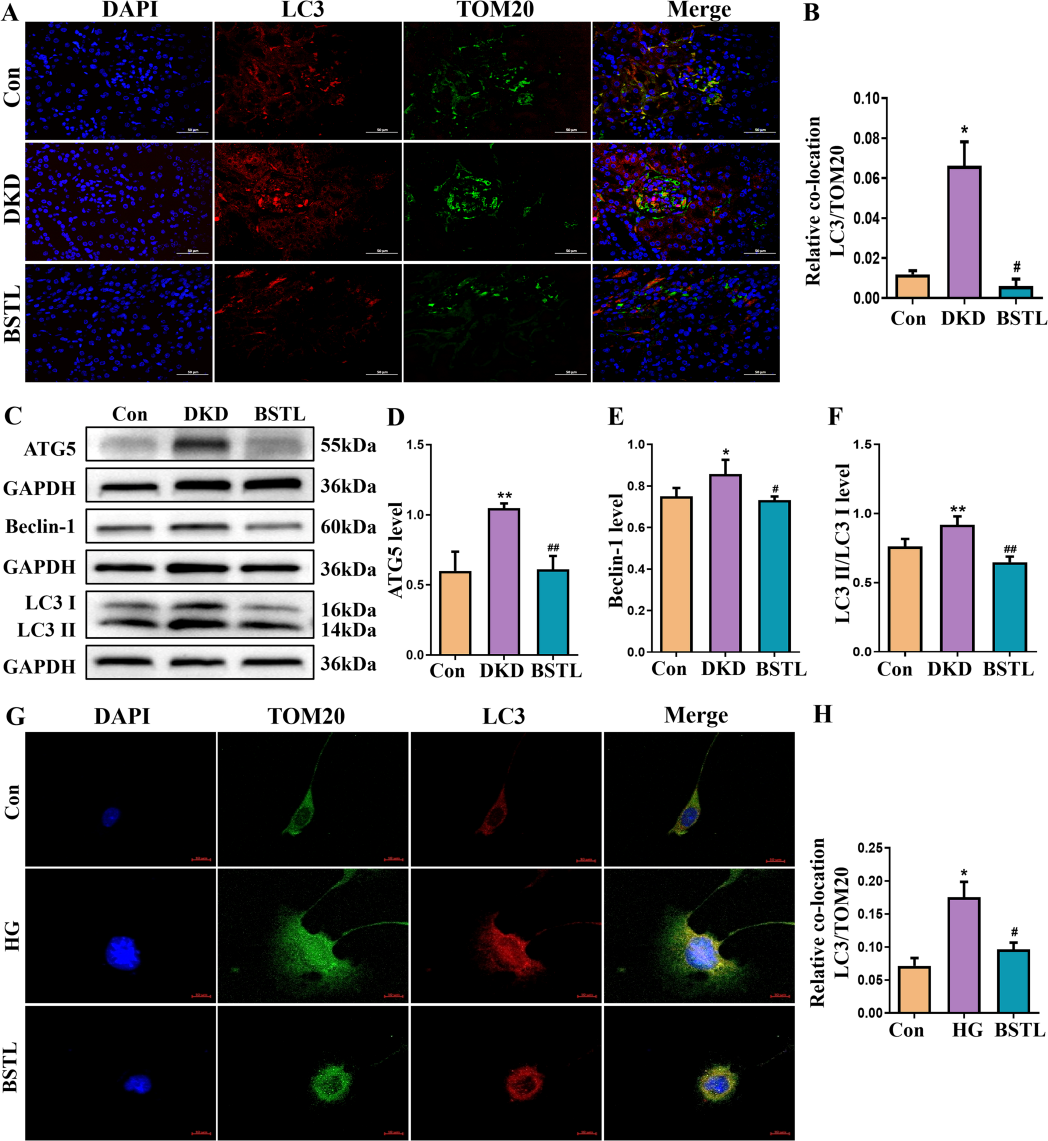
BSTL treatment reduced the expression of mitophagy-related proteins in the renal tissues of db/db mice and HG-treated podocytes. **(A)** Representative images of immunofluorescent co-localization staining of glomerular LC3 and TOM20 in different mice (×200). **(B)** Quantitative assessment of co-localization staining of glomerular LC3 and TOM20 in the kidneys of mice. **(C)** Representative western blot of ATG5, Beclin-1 and LC3 in the kidneys of mice. **(D-F)** Quantitative assessment of ATG5, Beclin-1 and LC3 in the kidneys of mice. *P<0.05 *vs*. con; **P<0.01 *vs*. Con; #P<0.05 *vs*. DKD; ##P<0.01 *vs*. DKD. con, control mice; DKD, db/db mice; BSTL, db/db mice treated with BSTL. **(G)** Representative images of immunofluorescent co-localization staining of LC3 and TOM20 in different cells (×200). **(H)** Quantitative assessment of co-localization staining of LC3 and TOM20 in different cells. *P<0.05 *vs*. con; #P<0.05 *vs*. HG. con, normal glucose; HG, high glucose; BSTL, high glucose combined with BSTL drug-containing serum.

A stable mitochondrial energy supply is essential to maintain the structural and functional integrity of podocytes. Mitophagy, a critical process for mitochondrial homeostasis, plays a complex role in the progression of DKD, acting as a double-edged sword. To further examine the impact of BSTL on mitophagy in DKD, we conducted immunofluorescence colocalization staining to assess LC3 and TOM20 expression. TOM20 was labeled with green fluorescence, LC3 with red fluorescence, and nuclei with DAPI (blue), with mitophagy indicated by yellow fluorescence resulting from the colocalization of LC3 and TOM20. As shown in **Figures 3A, B**, db/db mice exhibited greater colocalization of LC3 and TOM20 in the glomeruli compared to control mice, suggesting enhanced mitophagy in DKD. Notably, BSTL treatment significantly reduced this mitophagy level. We examined the expression of key mitophagy-related proteins to investigate the molecular mechanisms by which BSTL modulated mitophagy in podocytes. The results showed that the ATG5, Beclin-1, LC3 I, and LC3 II expression levels and the LC3 II/LC3 I ratio were elevated in db/db mice compared to controls. However, BSTL treatment reversed these alterations, indicating a regulatory effect on mitophagy (**Figures 3C–F**).

To further confirm the changes in mitophagy in podocytes, we performed immunofluorescence colocalization using MPC-5 cells from different experimental groups. LC3 was labeled with red fluorescence, TOM20 with green fluorescence, and nuclei with DAPI (blue). As shown in **Figures 3G, H**, MPC-5 cells cultured under high glucose conditions displayed an increased colocalization of LC3 and TOM20 compared to control cells, indicating an increase in mitophagy. However, BSTL-mediated serum treatment significantly reduced mitophagy levels, consistent with the findings observed in the animal model.

### 3.4 BSTL restored mitophagy in db/db mice and HG-cultured podocytes via the PINK1/Parkin signaling pathway

**Figure 4:**
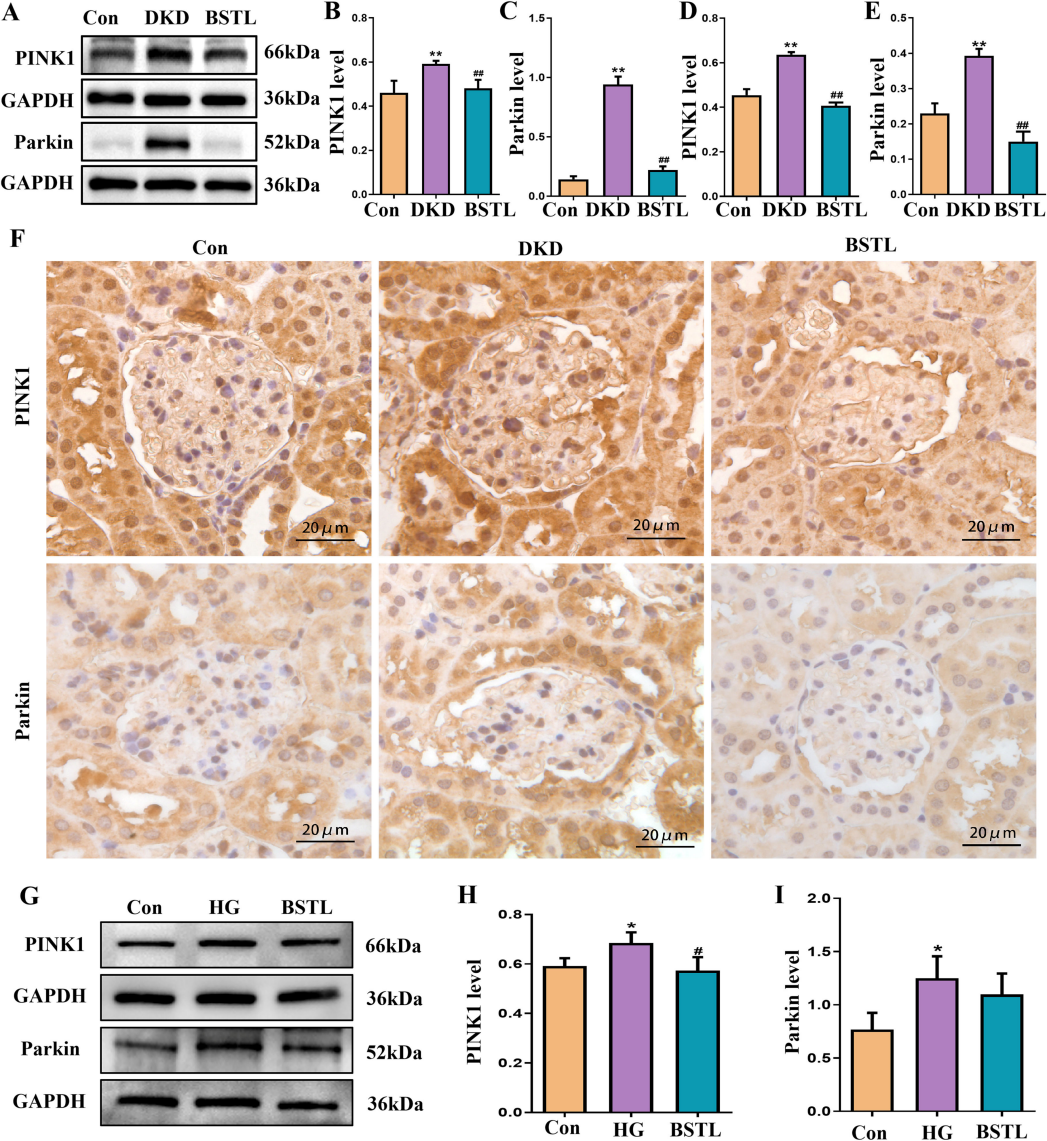
BSTL restored mitophagy in db/db mice and HG-cultured podocytes via the PINK1/Parkin signaling pathway. **(A)** Representative Western blots of PINK1 and the Parkin protein in the kidneys of mice. **(B, C)** Quantitative assessment of PINK1 and Parkin protein in the kidneys of mice. **(D, E)** Quantitative assessment of IHC staining of renal sections for the PINK1 and Parkin protein in different groups. **(F)** IHC staining of renal sections for PINK1 and Parkin protein in different groups (×400). *P<0.05 *vs*. con; **P<0.01 *vs*. Con; #P<0.05 *vs*. DKD; ##P<0.01 *vs*. DKD. con, control mice; DKD, db/db mice; BSTL, db/db mice treated with BSTL. **(G)** Representative Western blot and quantitative assessment of PINK1 and Parkin in podocytes. **(H, I)** Quantitative assessment of PINK1 and Parkin in podocytes. *P<0.05 *vs*. con; #P<0.05 *vs*. HG. con, normal glucose; HG, high glucose; BSTL, high glucose combined with BSTL drug-containing serum.

The PINK1/Parkin signaling pathway is a key regulator of mitophagy. To investigate whether BSTL modulates mitophagy to delay the progression of DKD through this pathway, we assessed the protein levels of PINK1 and Parkin in renal tissues by Western blotting. In db/db mice, both PINK1 and Parkin expression were significantly elevated, but these changes were markedly reversed after BSTL treatment (**Figures 4A–C**). To further examine the localization of these proteins in the glomeruli, we performed immunohistochemistry and observed consistent results (**Figures 4D–F**). *In vitro*, high-glucose treatment also led to increased expression of PINK1 and Parkin in MPC-5 cells, while BSTL-mediated serum treatment effectively downregulated the levels of both proteins (**Figures 4G–I**).

### 3.5 PINK1 deficiency in MPC-5 cells alleviated podocyte injury under HG ambience

**Figure 5:**
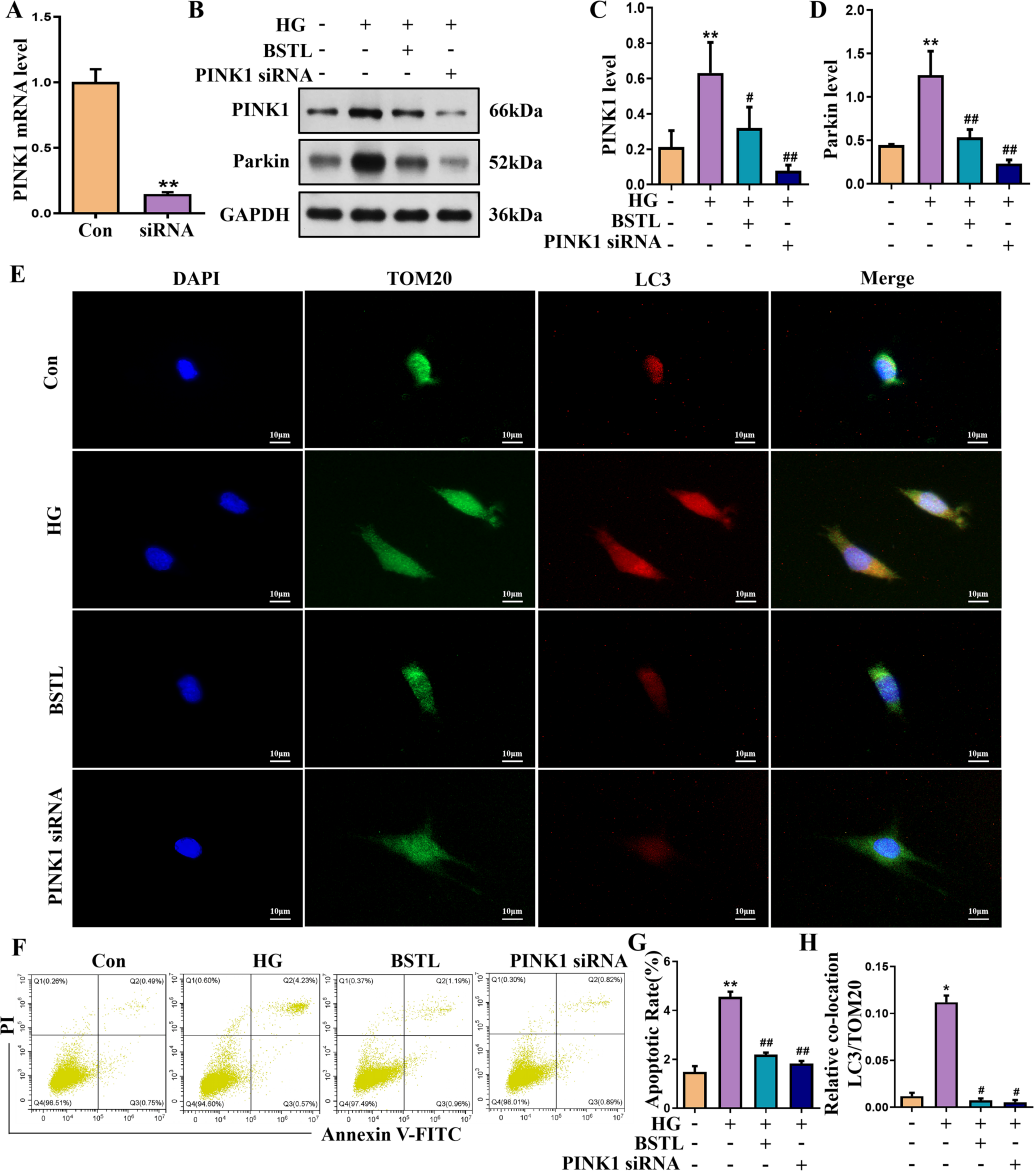
PINK1 deficiency in MPC-5 cells alleviates podocyte injury under the HG environment. **(A)** Quantitative assessment of the PINK1 gene in podocytes. **(B-D)** Representative Western blots of the PINK1 and Parkin protein in podocytes. **(E)** Representative images of immunofluorescent co-localization staining of LC3 and TOM20 in different cells (×200). **(F)** Representative flow cytometry analysis depicting the apoptosis detection in podocytes with different treatments (Q2 represented the ratio of late apoptotic cells, Q3 represented the ratio of early apoptotic cells, Q2+Q3 represented the total ratio of apoptotic cells). **(G)** Quantitative data expressing the overall percentage of podocyte apoptosis. **(H)** Quantitative assessment of co-localization staining of LC3 and TOM20 in different cells. *P<0.05 *vs*. con; #P<0.05 *vs*. HG. con, normal glucose; HG, high glucose; BSTL, high glucose combined with BSTL drug-containing serum; PINK1 siRNA, silenced PINK1 in HG condition.

We silenced PINK1 in podocytes by transfecting them with a specific siRNA targeting PINK1, resulting in a marked decrease in PINK1 expression, as shown in **Figure 5A**. PINK1 and Parkin protein expression was significantly reduced in PINK1 siRNA-treated podocytes compared to the model group in a high-glucose environment (**Figures 5B–D**). Next, we performed immunofluorescence co-labelling of LC3 and TOM20 in each group of cells and showed that knockdown of PINK1 reduced the level of co-localization of LC3 and TOM20 in podocytes cultured in high glucose (**Figures 5E, H**). Additionally, inhibition of PINK1 expression attenuated high glucose-induced podocyte damage, as confirmed by the percentage of apoptotic cells (**Figures 5F, G**).

## 4 Discussion

TCM, or ethnomedicine, has long accumulated valuable clinical experience and has been widely recognized in the prevention and treatment of various diseases or aberrant physiological environments. TCM showed significant benefits in preventing and treating renal diseases, including DKD. *Astragalus membranaceus* is one of the most important herbs in the BSTL Formula. Increasing evidence has shown that *Astragalus membranaceus* and its main chemical components, such as formononetin, hederagenin, and calycosin, could prevent and treat renal diseases, including DKD, by inhibiting endoplasmic reticulum stress, mitochondrial damage, long non-coding RNA A330074k22Rik/Axin2/β-catenin signaling pathway and ferroptosis (23–27). *Salvia miltiorrhiza* Bunge and *Rehmannia glutinosa*, as well as their bioactive compounds such as tanshinone IIA and catalpol, were also widely demonstrated to attenuate renal injury by a variety of underlying molecular mechanisms (28–33). Our current study demonstrates that the BSTL Formula mitigates podocyte damage in DKD by regulating mitophagy through the PINK1/Parkin signaling pathway.

Filtration slits connect the foot processes of podocytes through the slit diaphragm, which plays a critical role in establishing the selective permeability of the glomerular filtration barrier, preventing proteins from entering the urine (34). A persistent high-glucose environment induces podocyte apoptosis through various mechanisms. Analysis of biopsy samples from patients with type 1 and 2 diabetic glomerulosclerosis showed that the number of podocytes decreased proportionally with the severity of the injury and the degree of albuminuria and that it predicted disease progression. In contrast, albuminuria is one of the best predictors of decreased eGFR in DKD patients (35). The correlation between podocyte injury, proteinuria, and glomerulosclerosis suggests that podocyte injury is crucial to developing DKD. We observed an increase in apoptotic cells within the glomeruli of kidney tissues of *db/db* mice. Additionally, our *in vitro* assays yielded similar results, providing additional evidence to highlight the key role of podocyte injury in DKD progression.

Mitochondria are dynamic organelles with many functions critical for cellular metabolism and survival that participate in necrotic cell death and programmed apoptosis (36, 37). The kidney is one of the most energy-demanding organs in the human body (38) and has the second highest amount of mitochondria and oxygen consumption after the heart (39). Several studies have shown that *Astragalus membranaceus*, *Rehmannia glutinosa* and *Salvia miltiorrhiza* Bunge involved in BSTL could regulate mitochondria functions in renal disease (26, 40–42). Recent research has indicated that the kidney has a higher rate of mitophagy than other organs, underscoring its pivotal role in maintaining mitochondrial homeostasis (43) Mitophagy serves the crucial function of preserving mitochondria under optimal conditions by eliminating surplus or dysfunctional organelles (44–46), which is considered a protective mechanism under pathological conditions. Research also indicates that when mitochondrial damage remains within compensatory thresholds, the activation of mitochondrial autophagy can efficiently mitigate mitochondrial injury caused by external triggers. For example, recent publications have revealed that astragaloside II ameliorated podocyte injury and activated mitophagy in diabetic rats (47). Still, it prolonged or excessive mitophagy does not double this protective effect, and damages normal mitochondria, precipitating podocyte injury in the kidney and speeding the development of DKD (48). An earlier study has reported that *Astragalus membranaceus* and *Salvia miltiorrhiza* Bunge decoction attenuated DKD by inhibiting mitophagy in db/db mice (49). In our study, we also found that the expression of mitophagy-associated proteins, includingTOM20 and LC3 co-localization, ATG5, Beclin-1, and LC3, was upregulated in the db/db mice and high-glucose-cultured podocytes, indicating that mitophagy was enhanced in DKD, which means that excessive activation of mitophagy may also leads to phagocytosis and degradation of normal mitochondria by misrecognition, promoting the progression of DKD. Our findings further suggest that BSTL alleviates podocyte injury in DKD by inhibiting excessive mitochondria activation.

The most well-documented mechanism for mitophagy is the PINK1/Parkin pathway (50, 51). PINK1 is a mitochondrial protein kinase with a mitochondrial targeting sequence (MTS) and a transmembrane region (TMD) (52), which is usually located in the cytoplasm. Loss of mitochondrial membrane potential prevents PINK1 from being imported into mitochondria and induces the accumulation of PINK1 on the outer mitochondrial membrane (OMM) after mitochondrial damage (53). Then, PINK1 on the OMM recruits the cytosolic E3 ubiquitin-protein ligase Parkin to the damaged OMM and activates Parkin by direct phosphorylation and ubiquitination (17, 53). Active Parkin builds ubiquitin chains on OMM proteins, such as Mfn1/2, Mul1, and March5, which act as “autophagy” signals. The ubiquitin-labeled mitochondria are then identified by autophagy receptor proteins, such as Sequesterome 1 (SQSTM1/p62), Nuclear domain 10 protein 52 (NDP52), Optineurin (OPTN), Next to BRCA1 gene 1 (NBR1) and Tax 1 binding protein 1 (TAX1BP1), which connect mitochondria to autophagosomes through interaction with LC3, initiating the autophagic engulfment of mitochondria (54, 55). When autophagy is activated, the protein LC3-I is lipidated to form the protein LC3-II, which binds to the phosphatidylethanolamine on the autophagosome membrane and localizes to the intracellular autophagosome membrane. The PINK1/Parkin pathway may be involved in regulating podocyte damage and may play an important role in the development of DKD. Research has shown that PINK1/Parkin-mediated mitochondrial autophagy alleviates palmitic acid-induced podocyte apoptosis by reducing mitochondrial ROS generation (18). A previous study has shown that astragaloside IV suppresses aberrant activation of PINK1/Parkin-mediated mitophagy in *db/db* mice, thereby improving DKD (56). Our findings demonstrate that the PINK1/Parkin pathway is activated in high glucose-stimulated podocytes and *db/db* mice, with elevated expression of the mitochondrial autophagy marker LC3 and the autophagy-inducing proteins ATG5 and Beclin1. Moreover, the expression of the podocyte apoptosis-associated protein Bax was upregulated, while that of Bcl-2 was downregulated, indicating that high-glucose treatment induces the excessive activation of mitochondrial autophagy, promoting podocyte apoptosis. However, the BSTL intervention regulated the PINK1/Parkin signaling pathway, ameliorating excessive activation of mitochondrial autophagy in podocytes under DKD conditions and inhibiting podocyte apoptosis. Furthermore, studies also have demonstrated that astragaloside II upregulates the expression of PINK1 and Parkin associated with mitophagy and attenuate renal cell damage in diabetic rats (47). These findings suggest that the level of mitophagy in podocytes is a continuous dynamic adjustment process. Mitophagy exhibits varying responses across different experimental studies, potentially due to factors such as diverse animal models and disease stages (57).

In clinical practice, proteinuria is widely recognized as a critical risk factor for diabetic kidney disease (DKD) progression. Current therapeutic strategies primarily employ sodium-glucose cotransporter-2 (SGLT-2) inhibitors, glucagon-like peptide-1 (GLP-1) receptor agonists, and selective mineralocorticoid receptor antagonists (MRAs) to mitigate proteinuria and improve renal outcomes (58–61). However, the use of these medications remains limited in patients with severe renal impairment, and they carry risks of adverse effects including hyperkalemia and urinary tract infections, which restricts their broader clinical adoption (62).Traditional Chinese medicine (TCM) has demonstrated promising efficacy in slowing disease progression, alleviating symptoms, and reducing side effects. For instance, Niaoduqing granules have shown clinical benefits in DKD management (4). The Baoshentongluo formula, with its composition and dosages adhering to the safety thresholds specified in the Chinese Pharmacopoeia, represents another effective intervention for DKD. Notably, BSTL’s potential synergism with modern therapies warrants further exploration. A network meta-analysis revealed that combining Chinese patent medicines with ACEIs/ARBs enhances therapeutic efficacy and safety in early-stage DKD (20). Our future studies will systematically evaluate such combination strategies to maximize clinical benefits for DKD patients. In addition, the safety of drugs is very important for clinical application. While our murine studies confirmed BSTL’s short-term safety (no significant alterations in serum ALT and AST levels) (22), comprehensive long-term safety assessments remain essential for clinical translation.

## 5 Conclusion

We found abnormal activation of PINK1/Parkin-mediated mitophagy in DKD. Treatment with BSTL not only can reduce proteinuria levels and ameliorate renal pathological changes but also inhibit the overactivation of mitophagy to protect the podocytes in DKD.

## Data Availability

The original contributions presented in the study are included in the article/supplementary material. Further inquiries can be directed to the corresponding authors.
